# Research on multi-robot collaborative operation in logistics and warehousing using A3C optimized YOLOv5-PPO model

**DOI:** 10.3389/fnbot.2023.1329589

**Published:** 2024-01-23

**Authors:** Lei Wang, Guangjun Liu

**Affiliations:** ^1^School of Economy and Management, Hanjiang Normal University, Shiyan, Hubei, China; ^2^School of Business, Wuchang University of Technology, Wuhan, Hubei, China

**Keywords:** deep learning, multi-modal sensing, multi-robot collaboration, logistics automation, multi-agent systems, Warehouse robotics

## Abstract

**Introduction:**

In the field of logistics warehousing robots, collaborative operation and coordinated control have always been challenging issues. Although deep learning and reinforcement learning methods have made some progress in solving these problems, however, current research still has shortcomings. In particular, research on adaptive sensing and real-time decision-making of multi-robot swarms has not yet received sufficient attention.

**Methods:**

To fill this research gap, we propose a YOLOv5-PPO model based on A3C optimization. This model cleverly combines the target detection capabilities of YOLOv5 and the PPO reinforcement learning algorithm, aiming to improve the efficiency and accuracy of collaborative operations among logistics and warehousing robot groups.

**Results:**

Through extensive experimental evaluation on multiple datasets and tasks, the results show that in different scenarios, our model can successfully achieve multi-robot collaborative operation, significantly improve task completion efficiency, and maintain target detection and environment High accuracy of understanding.

**Discussion:**

In addition, our model shows excellent robustness and adaptability and can adapt to dynamic changes in the environment and fluctuations in demand, providing an effective method to solve the collaborative operation problem of logistics warehousing robots.

## 1 Introduction

In today's industrial automation field, logistics warehousing robots play a vital role and have become an indispensable part of logistics and warehousing management. With the rapid development of e-commerce and supply chain management, the demand and application of logistics warehousing robots are increasing (Liu et al., 2022). These robots are capable of automating multiple tasks, including cargo handling, inventory management, and order processing, to increase efficiency, reduce costs, and ease human burden.

However, achieving efficient logistics and warehousing operations puts forward higher requirements for robot collaborative operation and coordinated control to cope with dynamic changes in the environment and fluctuations in demand (Ning et al., 2022). Logistics warehousing robots often operate as multi-robot systems, which means they need to work closely together to complete tasks. In large-scale warehousing environments, collaborative operations involve complex issues such as path planning, task allocation, and dynamic scheduling (Huang et al., 2023). Collaborative operations between robots require a high degree of coordination and control to avoid collisions, improve efficiency, and ensure tasks are completed on time.

In recent years, deep learning technology has made significant progress in solving the collaborative operation and coordinated control problems of logistics and warehousing robots. Deep learning models, such as convolutional neural networks (CNN), reinforcement learning algorithms, and time series prediction methods, have been widely used to improve robots' perception, decision-making, and control capabilities (Zhang et al., 2023). Researchers have begun to explore how to use deep learning to optimize the operation of logistics warehousing robot swarms to achieve more efficient logistics management.

In this context, collaborative operation and coordinated control have become important areas in the research of logistics warehousing robots. This requires in-depth research on collaborative operation and coordinated control strategies to overcome these challenges and enable robots to work better together and improve overall efficiency and responsiveness (Tang et al., 2023). To achieve this goal, researchers have proposed and developed a variety of classic models and methods to solve the challenges of collaborative operation and coordinated control (Singh et al., 2023). These methods involve path planning, task allocation, and dynamic scheduling among robots to ensure efficient logistics warehousing operations. Therefore, research and application in the field of logistics warehousing robots will continue to benefit from the development of deep learning technology and collaborative operation and coordinated control strategies, which will help improve logistics efficiency, reduce costs, and meet growing logistics needs. Researchers have proposed and developed a variety of classic models and methods to solve the challenges of collaborative operation and coordinated control. Below are five commonly used related models.

Deep reinforcement learning models (Deep Reinforcement Learning, DRL) have made remarkable progress in recent years. It combines deep learning and reinforcement learning methods to enable logistics and warehousing robots to learn complex strategies to cope with challenges. The development of the DRL model can be traced back to the proposal of Deep Q-Network (DQN). This algorithm realizes the exploration and utilization of discrete action spaces by combining deep convolutional neural networks with Q-learning. Subsequently, the Deep Deterministic Policy Gradient (DDPG) algorithm introduced the processing of continuous action spaces, expanding the scope of application of DRL (Zhao et al., 2020). These algorithms provide new possibilities for autonomous learning and optimization strategies of logistics warehousing robots. DRL models are widely used in the field of logistics and warehousing robots, especially in tasks such as collaborative operations, path planning, and dynamic scheduling (Tang et al., 2019). These models allow robots to learn adaptive strategies in uncertain and complex environments, and have strong generalization capabilities. However, it is worth noting that the training process of the DRL model requires a large amount of data and computing resources, and may perform unstable in the initial stage. These problems require in-depth research and solution.

Multi-Agent Reinforcement Learning (MARL) focuses on collaborative decision-making and interaction between multiple agents. The development history of the MARL model is rooted in game theory, and many algorithms have subsequently emerged, including distributed PPO (Distributed PPO) and so on (Liang et al., 2021). These algorithms perform well in solving multi-robot cooperative operation and coordinated control problems. Among these problems, cooperation and competition between multiple agents are involved. The MARL model can well model the cooperative relationship between robots, thereby achieving better collaborative work. However, training MARL models requires considering complex interactions between agents, which increases computational complexity (Yu et al., 2022). Despite this, MARL is still one of the important tools to solve the cooperative operation and coordinated control problems of multi-robots. It provides a powerful framework for team collaborative actions of logistics warehousing robots.

Classical Path Planning Algorithms such as A^*^ and Dijkstra have a profound historical background and have played an important role in the fields of automatic navigation and robot path planning since their birth. These algorithms were mainly used in the path planning of single robots in the early days. Their core advantage lies in their ability to efficiently avoid collisions and find optimal paths (Liang et al., 2022). Dijkstra's algorithm is known for its wide applicability and reliability and is widely used in fields such as map navigation and network routing. The A^*^ algorithm provides more efficient path planning capabilities while taking heuristic search into account. However, although these classical path planning algorithms perform well in small-scale tasks, they usually ignore the need for cooperative operations among multiple robots (Sahu and Das, 2022). In large-scale scenarios where logistics and warehousing robot groups operate collaboratively, the application of this single-robot path planning algorithm is limited. In multi-robot systems, robots need to work together to achieve task allocation, avoid conflicts, and optimize resource utilization, and traditional path planning algorithms are not sufficient to solve these challenges.

Distributed Control Systems are a control strategy widely used in many fields, including manufacturing, industrial automation, and logistics management (Eudes et al., 2023). The core feature of these systems is the use of multiple controllers working simultaneously to complete tasks collaboratively. Distributed control systems show strong advantages in tasks that require coordinated operation and control among multiple robots. Its core mechanism is to decompose complex tasks into multiple relatively simple subtasks and work together to complete the entire task efficiently, thus improving efficiency and performance. In the field of logistics and warehousing, the application of distributed control systems is particularly significant (Gindullina et al., 2021). Groups of logistics and warehousing robots need to work together to achieve efficient execution of tasks, such as cargo handling, inventory management, and order processing. Distributed control systems can allocate these tasks to different robots while coordinating their work to ensure overall system efficiency. This approach allows robots to better adapt to changing needs and environmental conditions. However, to achieve effective distributed control, good communication and coordination strategies are required. Robots need to be able to share information in real time, including task status, environmental changes, and actions of other robots. This requires reliable communication infrastructure and protocols to ensure accurate delivery and synchronization of information. In addition, the design of collaborative strategies is also a key issue. Researchers need to consider how to allocate tasks, how to handle conflicts, and how to optimize resource utilization to make distributed control systems work best in complex multi-robot environments.

The time series forecasting problem has always been a classic challenge in the field of time series analysis. Many traditional methods and deep learning methods have emerged in this field, the most well-known of which include recurrent neural network (RNN) and long short-term memory network (LSTM) (Jiang et al., 2024). These methods play a key role in time series data analysis in different fields, and can also play an important role in the collaborative operation and coordinated control of logistics warehousing robot groups. These methods can handle various types of time series data, such as cargo flow, demand changes, robot positions, etc., providing important support for robot decision-making. The application of time series prediction models is particularly important in the field of logistics and warehousing. In warehouse management, accurately predicting the flow of goods and changes in demand is a key factor in maintaining high efficiency and meeting customer needs. For example, by accurately predicting the flow of goods, robots can plan paths in advance, reducing goods handling time and energy consumption. The prediction of demand changes can help robots better allocate tasks and resources to cope with changing needs. However, the accuracy of time series forecasting is often affected by data quality and model selection (Alqudah et al., 2022). Data quality issues include missing data, outliers, and noise, which may cause the model's predictive performance to degrade. Therefore, data preprocessing and cleaning are crucial steps. In addition, model selection is also a key issue. Traditional time series forecasting methods such as Autoregressive Moving Average Model (ARIMA) are usually able to cope with general problems, but may not perform well when dealing with non-linear, non-stationary or high-dimensional data. Deep learning methods such as RNN and LSTM have stronger fitting capabilities, but also require a large amount of training data and computing resources.

The above are five commonly used models in collaborative operation and coordinated control of logistics warehousing robot groups: deep reinforcement learning model (DRL), multi-agent reinforcement learning model (MARL), classic path planning algorithms (Classical Path Planning Algorithms), distributed Control systems (Distributed Control Systems) and time series prediction models (Time Series Prediction Models). Although these models have achieved certain achievements in different situations, they also have some shortcomings. First, when dealing with collaborative operations of logistics warehousing robots, deep reinforcement learning models (DRL) may require a large amount of training data and computing resources, and their performance is unstable in the initial stage. Although the multi-agent reinforcement learning model (MARL) can model the cooperative relationship between robots, it needs to consider complex agent interactions and has high computational complexity. Although classic path planning algorithms are efficient and deterministic, they usually do not consider collaborative operations between robots, limiting their application in large-scale multi-robot systems. Distributed control systems excel in collaborative operation and control tasks but require effective communication and collaboration strategies. Time series forecasting models are affected by data quality and model selection in terms of forecast accuracy.

Based on the shortcomings of the above work, we proposed the YOLOv5-PPO model and enhanced it with the A3C optimization algorithm. The YOLOv5-PPO model combines fast real-time target detection capabilities (YOLOv5) and reinforcement learning algorithms (PPO) to deal with path planning and dynamic scheduling problems of logistics warehousing robots. First of all, YOLOv5 is partially responsible for real-time target detection and environment awareness. It is able to identify and track objects to obtain real-time environmental information. The role of this section is to provide accurate sensory data for the robot to better understand the environment. Secondly, the PPO part is responsible for real-time decision making. It uses reinforcement learning algorithms to generate the robot's action strategy based on the current state. PPO is able to learn adaptive strategies in uncertain and complex environments to ensure that robots can operate efficiently together. Finally, the A3C optimization algorithm is used to improve the overall model performance. It can train the YOLOv5-PPO model in parallel, thereby speeding up training and improving performance. A3C can coordinate the movements of multiple robots to achieve more efficient collaborative operations. Compared with previous models, the model we constructed has important significance and advantages. First, it enables adaptive sensing and real-time decision-making to adapt to dynamic changes in the environment and fluctuations in demand. Secondly, the model combines target detection and reinforcement learning to have stronger perception and decision-making capabilities. Most importantly, our model is optimized using the A3C algorithm, which improves training efficiency and performance, allowing robots to operate better together. This will help promote the research and application of collaborative operation and coordinated control of logistics warehousing robot groups.

The main contributions of this study are as follows:

1. Proposition of new model. We introduced the YOLOv5-PPO model, which combines real-time target detection (YOLOv5) with a reinforcement learning algorithm (PPO) to achieve collaborative operation of robot perception and decision-making. This model is novel in the field of logistics and warehousing and can provide multi-robot systems with more powerful adaptive perception and real-time decision-making capabilities.
2. Application of A3C algorithm. We used the Asynchronous Advantage Actor-Critic (A3C) algorithm to optimize the YOLOv5-PPO model, improving the model's performance and training efficiency. The parallel training of the A3C algorithm enables the model to adapt to changes in the environment and fluctuations in demand more quickly, thereby improving the efficiency of collaborative operations.
3. Promote the development of logistics and warehousing robots. Our research is not only of great theoretical significance, but is also expected to promote practical applications in the field of logistics warehousing robots. By providing more advanced perception and decision-making capabilities, our model is expected to promote collaborative operation and coordinated control of logistics warehousing robot groups, thereby improving the efficiency and reliability of logistics operations.

In the following article structure, we will organize the content in the following way: In Section 2, we will comprehensively review relevant research and literature in the field of collaborative operation and coordinated control of logistics warehousing robot swarms. Section 3 will introduce in detail the key details of our proposed YOLOv5-PPO model and the A3C optimization algorithm. Section 4 will focus on our experimental design and experimental results. Finally, Section 5 will be the summary and discussion of this study.

## 2 Related works

### 2.1 Traditional method

The collaborative operation and coordinated control of logistics warehousing robots is a complex and critical issue involving cooperation and interaction between multiple robots. Traditional logistics warehousing robot control methods have a long history in this field, including classic path planning algorithms, distributed control systems, and traditional task allocation and scheduling. However, the application of these methods in large-scale multi-robot systems is limited, requiring more adaptable and flexible methods to cope with changing needs and environments.

Classic path planning methods include A^*^ algorithm, Dijkstra algorithm, and other heuristic search methods. Their development can be traced back to the mid-20th century. These algorithms find the shortest path or optimal solution by searching nodes in the space to avoid collisions and plan the robot's trajectory. The advantage of these classical methods is their efficiency and determinism (Binder et al., 2019). They perform well in small-scale tasks and have been widely used in fields such as automatic navigation, path planning, and logistics warehousing. However, these methods also face some challenges. First, they usually do not consider collaborative operations between robots and are difficult to adapt to the complex environment of large-scale multi-robot systems. Secondly, these algorithms perform poorly when dealing with dynamic environments and demand fluctuations, lacking flexibility and adaptability.

Distributed control system is a control strategy that has been widely used in many fields. Their development can be traced back to the 1970s. These systems use multiple controllers to work together and are suitable for tasks that require coordinated operation and control between multiple robots. Distributed control systems can decompose tasks into multiple subtasks and complete them by working together, thereby improving efficiency. However, to ensure synchronization between various controllers, effective communication and collaboration strategies are required. This is a challenge for large-scale multi-robot systems because the complexity of interactions between robots increases the complexity of communication (Salimi et al., 2022). In addition, distributed control systems often require carefully designed coordination strategies, which makes them difficult to cope with dynamic environments and demand changes.

Traditional task allocation and scheduling methods are one of the commonly used strategies in logistics warehousing robots. These methods were initially used in the manufacturing field, and then gradually expanded to the logistics and warehousing fields. Their development can be traced back to the 1990s. These methods mainly include rules-based task allocation, shortest task time (SPT) and other algorithms. These methods still have some applications in small-scale tasks because they are deterministic and controllable (Deng et al., 2023). However, they usually require static planning in advance and are difficult to cope with dynamic environments and demand fluctuations. Traditional task allocation and scheduling methods lack adaptability and are difficult to cope with collaborative operations and control between robots, so their efficiency in large-scale multi-robot systems is limited.

### 2.2 Reinforcement learning

The application of reinforcement learning in the field of logistics and warehousing robots has made significant progress. Reinforcement learning is a machine learning method designed to enable an agent to learn how to take actions to maximize cumulative rewards through interaction with the environment. In the field of logistics and warehousing robots, reinforcement learning is widely used in tasks such as path planning, task allocation, dynamic scheduling and collaborative operations. Among them, deep reinforcement learning (DRL) is a method that combines deep learning and reinforcement learning and has achieved great success. DRL models such as Deep Q-Networks (DQN) and Deep Deterministic Policy Gradients (DDPG) have achieved significant breakthroughs in the field of robotics.

The advantage of the DRL model is its ability to handle high-dimensional state space and action space, enabling robots to learn complex strategies. They also have strong generalization capabilities and can adapt to changing environments and task requirements (Han et al., 2022). However, the training of DRL models usually requires a large amount of data and computing resources, and the performance may be unstable in the initial stage. In addition, the interpretability of the DRL model is relatively poor, making it difficult to understand its decision-making process. In addition, multi-agent reinforcement learning (MARL) is an important method to solve the cooperative operation and coordinated control problems of multi-robots. The MARL model focuses on collaborative decision-making and interaction between multiple agents (Hu et al., 2023). Its development originates from game theory and distributed optimization algorithms, such as distributed PPO (Distributed PPO) and so on. The MARL model can model the cooperative relationship between robots so that they can work better together. However, training MARL models requires considering complex interactions between agents, and the computational complexity is high.

Although reinforcement learning has made significant progress in the field of logistics and warehousing robots, it still faces some important limitations and challenges. First, DRL models require a large amount of data and computing resources to train, which may be impractical for real-world logistics warehousing systems. In addition, the training process of DRL models can be very time-consuming and requires effective acceleration in practical applications.

Another challenge is the interpretability issue of DRL models. Due to the complexity of its deep learning components, the decision-making process of DRL models is often difficult to interpret, which may be limiting in applications that require transparency and interpretability. In terms of MARL, complex interactions and collaborative decision-making between multiple agents make model training and tuning more complex. The computational complexity of MARL models increases the size of the problem, resulting in a significant increase in training time.

### 2.3 Time series forecasting

The application of time series prediction in the field of logistics and warehousing robots is closely related to task scheduling and collaborative operations. Time series forecasting aims to predict values or trends at future time points based on historical data. In logistics warehousing, this can be used to predict the arrival of goods or changes in demand to better schedule the work of robots (Park et al., 2022). This section describes the development of time series forecasting, classical models, and the advantages and limitations of these models.

The development of time series forecasting can be traced back many years, when statistical methods such as moving averages and exponential smoothing were widely used (Orr and Dutta, 2023). However, with the rise of machine learning and deep learning, traditional methods are gradually being replaced by more powerful models. In the field of logistics warehousing robots, classic time series models include autoregressive models (AR), moving average models (MA), autoregressive integrated moving average models (ARIMA), seasonal Decomposition methods, etc. These models model based on historical data of a time series and are generally suitable for stationary time series. They have some predictive accuracy but perform poorly when dealing with non-linear and non-stationary data.

With the development of deep learning, models such as Recurrent Neural Networks (RNN) and Long Short-Term Memory (LSTM) have become emerging choices for time series prediction. These deep learning models are better able to capture nonlinear relationships and long-term dependencies in time series, and are suitable for complex and non-stationary time series data (Almadhoun et al., 2021). In the field of logistics and warehousing robots, these models have been successfully used in tasks such as predicting the flow of goods and predicting demand changes. Their advantage is that they can handle multi-modal sensing data and have high prediction accuracy.

Although time series prediction has been widely used in the field of logistics and warehousing robots, it still faces some limitations and challenges. First, the accuracy of time series forecasting models is highly dependent on the quality and quantity of historical data. If historical data is insufficient or contains a lot of noise, forecast results may be unstable. Therefore, the quality of data collection and processing is crucial. Secondly, time series models usually assume that data are stationary, but in actual logistics and warehousing, data are often non-stationary and are affected by seasonality, trend and periodicity. This makes model building and prediction more complex. In addition, time series models may not capture sudden events or anomalies, so consideration needs to be given to how to handle these uncertainties. Finally, time series forecasting usually provides predictions of future trends, but how to apply these trends to actual decision-making of robots to achieve collaborative operation and coordinated control still requires further research.

### 2.4 Multimodal perception and deep learning

The development of multi-modal perception and deep learning in the field of logistics and warehousing robots is one of the important factors promoting collaborative operation and coordinated control.

Multimodal perception means that the robot system obtains different types of information through a variety of sensors, such as vision, lidar, ultrasound, etc., to understand the environment more comprehensively. Significant progress has been made in the development of multimodal perception, enabling robots to perceive and understand complex environments (Efthymiou et al., 2022). In the field of visual perception, deep learning has made major breakthroughs in tasks such as object detection, image segmentation, and target tracking. Deep learning models such as Convolutional Neural Networks (CNN) and Convolutional Recurrent Neural Networks (CRNN) are widely used in visual perception. In addition, non-visual sensors such as lidar and ultrasonic sensors also play an important role in multi-modal perception.

The application of deep learning in multi-modal perception not only improves the processing capabilities of sensor data, but also enables robots to perform cross-modal information fusion. For example, by combining visual information and lidar data, more accurate environmental maps can be built. The advantage of multi-modal perception is that it can provide more comprehensive and reliable environmental perception to support the robot's decision-making and actions. In the field of logistics and warehousing robots, multi-modal perception can be used to identify goods, detect obstacles, monitor environmental temperature and humidity, etc., providing a wealth of information for collaborative operation of robots (Barisic et al., 2022).

In deep learning technology, especially in multi-modal perception, it achieves the fusion of cross-modal information while improving sensor data processing capabilities. This integration of information not only facilitates the construction of more accurate environmental maps, but also plays a key role in failure prediction. Through comprehensive analysis of multi-sensor data, possible sensor fault signals can be detected, enabling early detection and intervention of potential problems (Queralta et al., 2020). However, multimodal sensing faces a series of challenges in fault prediction. First, the introduction of multiple sensors may increase hardware costs and system complexity, requiring a balance between performance and feasibility in the design. Second, data fusion and calibration involve solving complex technical problems to ensure the accuracy of individual sensor data. Furthermore, training of deep learning models requires large amounts of labeled data and computing resources, and difficulties may be faced in obtaining faulty data.

## 3 Methodology

### 3.1 Overview of our model

In this study, we proposed a YOLOv5-PPO model based on Asynchronous Advantage Actor-Critic (A3C) optimization, aiming to realize multi-robot collaborative operation and coordinated control of logistics warehousing robots. The model integrates key technologies such as multi-modal perception, real-time decision-making and deep learning to adapt to dynamic changes in the environment and fluctuations in demand.

YOLOv5 is the basis of our model and is known for its fast real-time object detection capabilities. YOLOv5 can combine sensory information with time series data for real-time target detection and environment understanding. Compared with traditional target detection algorithms, YOLOv5 has higher detection speed and accuracy, and is suitable for multi-robot perception tasks in logistics and warehousing scenarios.

Proximal Policy Optimization (PPO) is another important component of our model. It is a reinforcement learning algorithm used for real-time decision-making problems and suitable for path planning and dynamic scheduling of logistics warehousing robots. The PPO algorithm generates action strategies by training agents to maximize cumulative rewards. In our model, the PPO algorithm is used for decision making to dynamically generate the robot's action strategy based on the current state of the model. This reinforcement learning method allows robots to learn and optimize strategies autonomously under different situations, improving the efficiency of collaborative operations.

The optimization algorithm Asynchronous Advantage Actor-Critic (A3C) is the key to integrating YOLOv5 and PPO into a unified framework. A3C is a distributed reinforcement learning algorithm that can be used to train deep learning models in parallel. In our model, the role of the A3C algorithm is to coordinate the actions of multiple robots to achieve more efficient collaborative operations. The A3C algorithm can train multiple agents at the same time, providing the advantages of parallel training and helping to accelerate model convergence and improve performance.

Throughout the model, YOLOv5, PPO, and A3C work together with each other. YOLOv5 provides perception information through real-time target detection, PPO generates decision-making strategies through reinforcement learning, and A3C coordinates the actions of multiple robots through distributed training. This comprehensive design enables our model to adapt to dynamic environmental changes while maintaining efficient collaborative operations and coordinated control.

Our model building process is divided into the following key steps: first, we collect and prepare multi-modal data such as sensory information, environmental status, robot position, and task requirements for multiple time steps. These data include images, sensor data, and time series information, which are preprocessed and used to train and test models. Then, we train based on the basic model of YOLOv5 for real-time target detection and environment perception. We use large-scale labeled data sets to perform supervised training on the YOLOv5 model to ensure high accuracy and robustness of target detection. Next, we use the Proximal Policy Optimization (PPO) algorithm to train the robot's action strategy for path planning and dynamic scheduling problems. During the training process, we use an environment simulator to simulate logistics warehousing scenarios and guide model learning based on the reward function. Finally, we integrate YOLOv5 and PPO into the framework of the A3C algorithm. The A3C algorithm is responsible for coordinating the actions of multiple robots and training in a parallel environment. This integrated framework allows multiple robots to simultaneously learn and optimize strategies to adapt to changes in the environment and fluctuations in demand.

**Figure 1:**
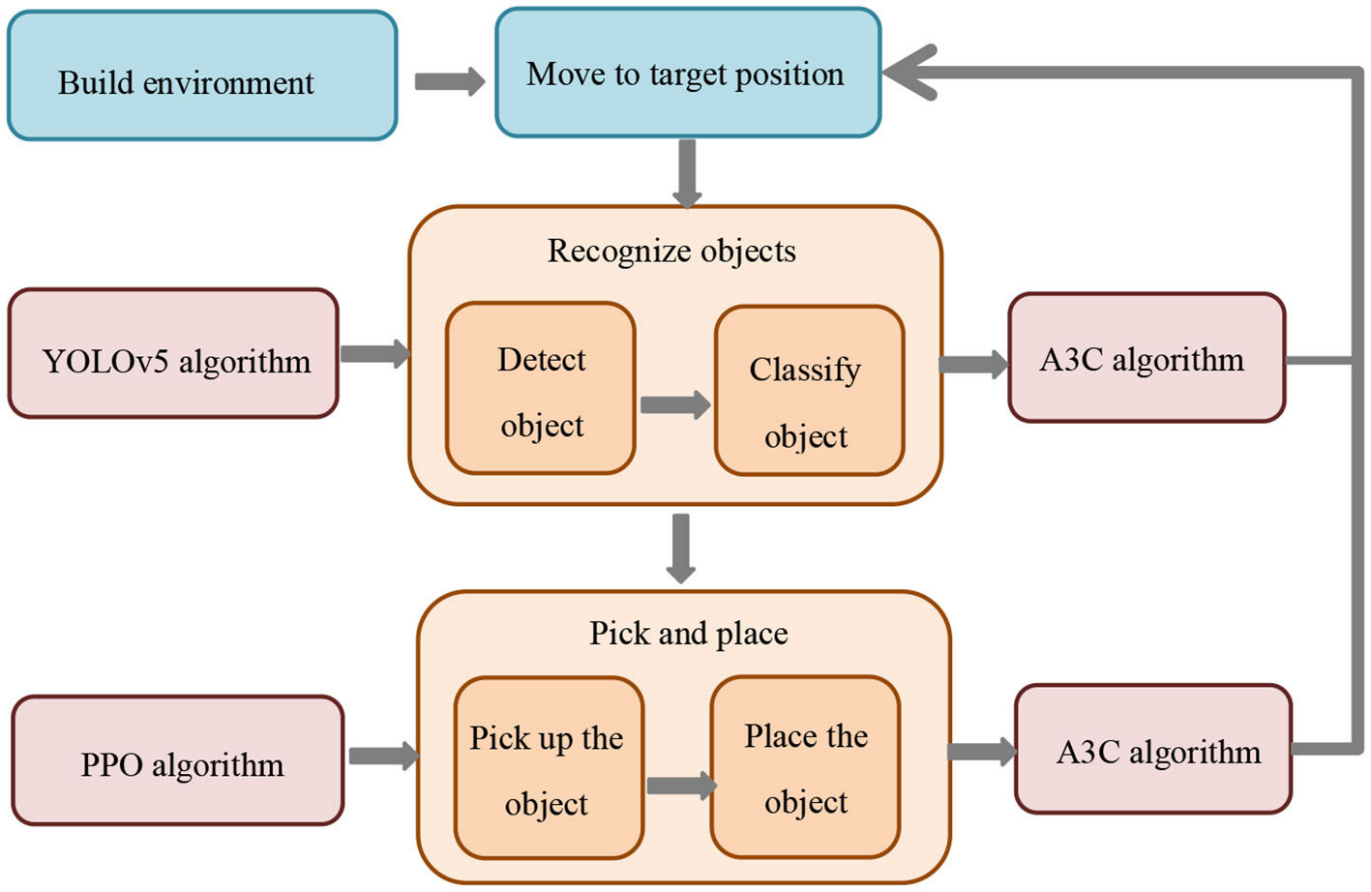
Overall flow chart of the model.

The structural diagram of the overall model is shown in Figure 1.

Our model enables adaptive sensing and real-time decision-making among multiple robots to adapt to dynamic changes in the environment and fluctuations in demand. This provides key support for the efficient execution of logistics and warehousing tasks and improves the overall efficiency of the logistics and warehousing system. The model integrates key technologies such as multi-modal perception, deep learning and reinforcement learning, which can perceive the environment more comprehensively and reliably, optimize decision-making strategies, and achieve collaborative operations among multiple robots. This not only improves the quality of task completion, but also improves the intelligence level of the robot system. In addition, this model has broad application prospects. It can not only be used in the field of logistics and warehousing, but can also be extended to other multi-robot collaborative operation scenarios, such as manufacturing and medical fields. It provides a general method for solving complex multi-robot collaboration problems.

The running process of the YOLOv5-PPO model is shown in Algorithm 1.

**Algorithm 1:**
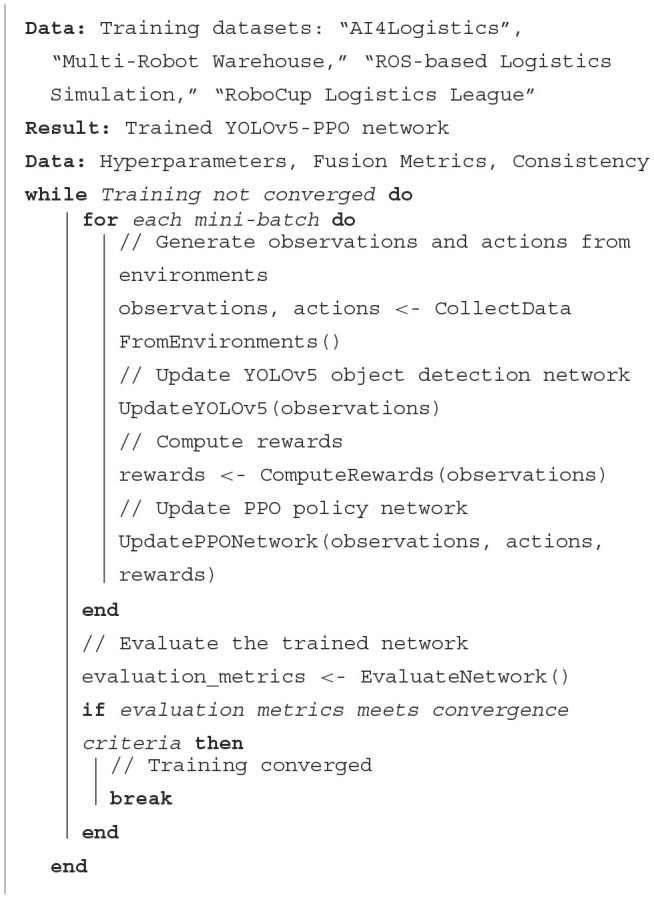
YOLOv5-PPO training.

### 3.2 YOLOv5 model

You Only Look Once version 5 (YOLOv5) is an advanced target detection model that uses a single-stage target detection algorithm to predict multiple target bounding boxes and categories in an image through one forward propagation. The core principles of the model include Anchor Boxes, Backbone Network, Detection Head, and Non-Maximum Suppression (Rong et al., 2023). Together, these elements ensure efficient and accurate target detection. YOLOv5 is widely used in the field of logistics and warehousing robots. Its fast inference speed enables the robot to perceive the environment in real time, while its high accuracy ensures the reliability of target detection. In multi-robot collaborative operations, accurate target detection is crucial to task allocation and path planning. YOLOv5's target detection results provide key input for subsequent decisions, allowing robots to work together more intelligently.

YOLOv5 is a key component of multi-robot collaborative operation of logistics warehousing robots. In a logistics warehousing environment, robots need to detect and track multiple types of goods and obstacles to complete tasks collaboratively. The target detection capability of YOLOv5 just meets this demand and provides the necessary visual perception support for multi-robot collaborative operations. YOLOv5 provides our overall model with efficient and accurate object detection capabilities. Its fast reasoning speed enables logistics warehousing robots to perform target perception and environment understanding in real-time environments. At the same time, the high accuracy of YOLOv5 ensures the reliability of target detection and helps improve the quality of task execution. In multi-robot collaborative operations, accurate target detection is crucial for task allocation and path planning. The target detection results of YOLOv5 provide important input for subsequent decision-making, allowing the robot to better understand the environment and make adaptive decisions.

YOLOv5 uses a multi-layer perception backbone network and advanced strategies to detect objects in images The following are the core mathematical formulas in YOLOv5, which show how the model performs object detection.

First is the Anchor Box Definition formula (1):


(1) $$\begin{array}{l} \text{Anchor boxes}={\left\{{\text{width}}_{\text{i}},{\text{height}}_{\text{i}}\right\}}_{\text{I}-1}^{\text{N}} \end{array}$$

In this formula, Anchor boxes define anchor boxes of different sizes and proportions for detecting targets of different sizes. *width*_*i*_ represents the width of the *i*-th anchor box, and *height*_*i*_ represents the height of the *i*-th anchor box.

Then there is the Bounding Box Prediction formula (2):


(2) $$\begin{array}{l} {\text{BBox}}_{\text{i,j}}\text{= }{\text{Sigmoid(tx}}_{\text{i,j}}\text{) + }{\text{c}}_{\text{x}}{\text{,Sigmoid(ty}}_{\text{i,j}}\text{) + }{\text{c}}_{\text{y}}{\text{,exp(tw}}_{\text{i,j}}\text{)}\\ \;{\text{·pw}}_{\text{i}}{\text{,exp(th}}_{\text{i,j}}{\text{)·ph}}_{\text{i}} \end{array}$$

In this formula, BBox_i, j_ represents the bounding box of the *i*-th anchor box at position j. tx_i, j_ represents the offset of the x coordinate. ty_i, j_ represents the offset of the *y* coordinate. tw_i, j_ represents the offset of the width. th_i, j_ represents the offset of the height. c_x_ and c_y_ represent the coordinates of the upper left corner of grid cell j. pw_i_ and ph_i_ represent the width and height of the *i*-th anchor box.

Finally, there is the Class Score Prediction formula (3):


(3) $${\text{P}}_{\text{i},\text{j}}(\text{class})=\text{Sigmoid}({\text{t}}_{\text{i},\text{j}}(\text{class}))$$

In this formula, P_i, j_(class) represents the score of the category class of the *i*-th anchor box at position j. t_i, j_(class) represents the predicted value of category class.

These formulas form the core components of the YOLOv5 model, and they are responsible for predicting the location, size, and object category of the bounding box. YOLOv5 trains these formulas to enable the model to accurately detect and locate objects in images. The anchor boxes and predicted values at different positions are related to each other to generate the results of target detection.

**Figure 2:**
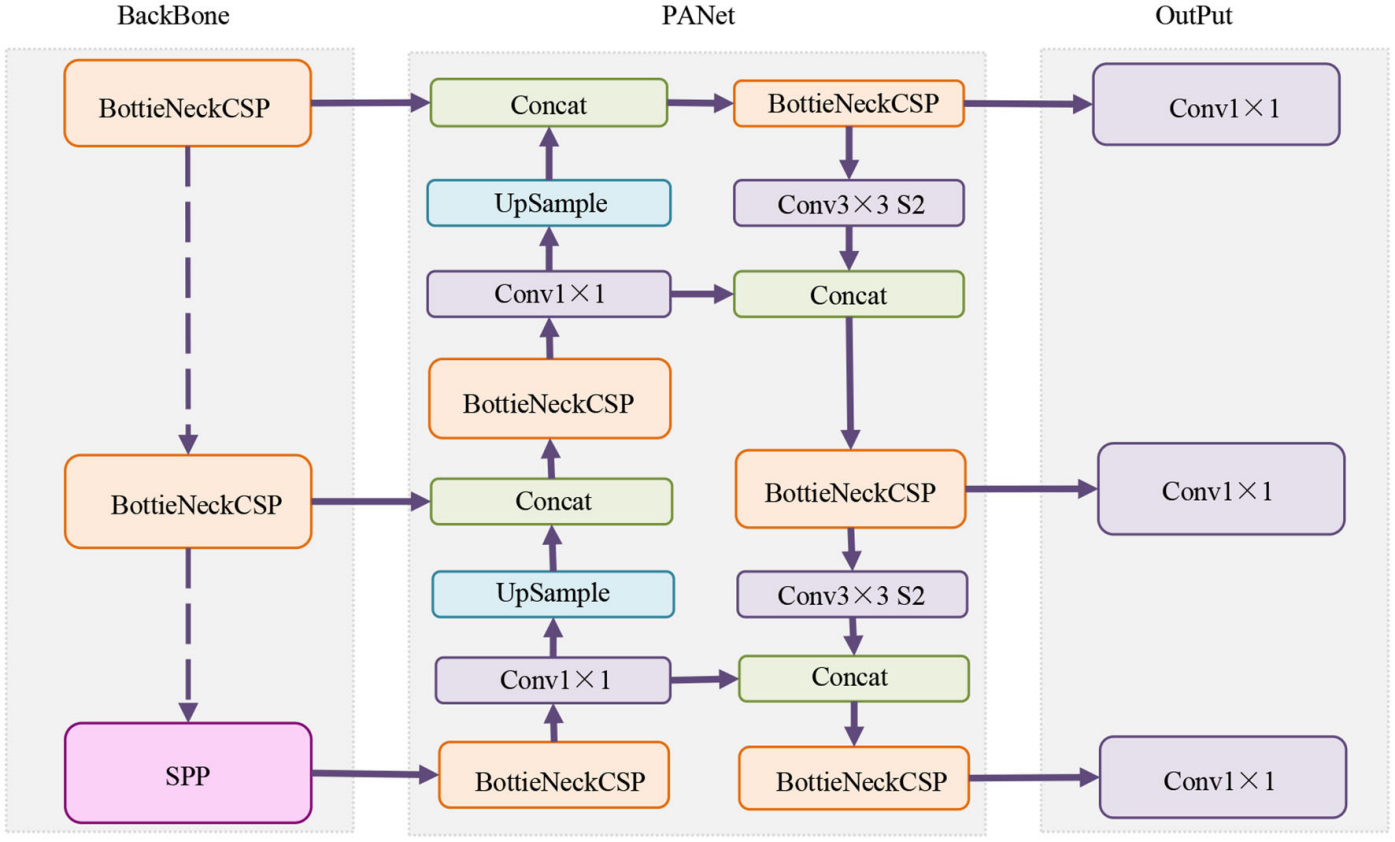
Flow chart of the YOLOv5 model.

The structure diagram of YOLOv5 Model is shown in Figure 2.

### 3.3 PPO model

Proximal Policy Optimization (PPO) is a reinforcement learning algorithm designed to solve real-time decision-making problems and shows excellent applicability in path planning and dynamic scheduling of logistics warehousing robots (Li et al., 2020). The core principle of this algorithm is to maximize the cumulative reward by optimizing the policy function, so that the robot learns to perform effective decisions.

The PPO model makes an important contribution to the decision-making and path planning stages of the overall model. In a group of logistics warehousing robots, real-time decision-making and path planning are crucial for multi-robot collaborative operations. The PPO model implements reinforcement learning through the Actor-Critic structure, that is, the two network structures of Actor and Critic. Actors are responsible for formulating the robot's action strategies, while Critics evaluate the value of these strategies. The PPO algorithm ensures that the policy changes will not be too large through proximal policy optimization, improving the stability and convergence speed of training. This feature enables the PPO model to dynamically generate optimization strategies for the robot to maximize task completion efficiency.

The PPO model is a key component of collaborative operation and control of logistics warehousing robots. Its advantage lies in processing high-dimensional state space and continuous action space, adapting to the needs of logistics and warehousing robots to perceive multiple targets and perform continuous motion in multi-robot collaborative operations. By optimizing the policy function, the PPO model provides efficient decision-making capabilities, allowing the robot to better adapt to dynamic changes in the environment and fluctuations in demand.

PPO adopts a cutting and pruning policy update method to ensure that policy changes are within an acceptable range to improve training stability. The following is the core formula of PPO and the corresponding variable explanations.

First, the objective function (4) of PPO is as follows:


(4) $$\begin{array}{l} \text{J}\left(\theta \right)=\text{IE}\left[\frac{\pi \left(\text{ a}|\text{s}\right)}{\pi_{\text{old}}\left(\text{ a}|\text{s}\right)}\text{A}\left(\text{s},\text{a}\right)\right]-\beta \text{IE}\left[\text{KL}\left(\pi_{\text{old}}\left(\cdot |\text{S}\right),\pi \left(\cdot |\text{S}\right)\right)\right] \end{array}$$

In this formula, J(θ) is the objective function used to update the parameters θ of the policy during training. π( a|s) represents the policy function, the probability of taking action a given state s.π_old_( a|s) represents the old policy function, which is used to calculate the KL divergence. A(s, a) is the advantage function that measures the advantage of taking action a relative to the average action. β is a hyperparameter that controls KL divergence. KL(π_old_(·|S), π(·|S)) represents the KL divergence between the policy distribution π_old_ and the new policy distribution π.

Second, PPO also introduces an alternative objective function (5), as follows:


(5) $$\begin{array}{l} \text{ L}(\theta )=\min\left(\frac{\pi (\text{ a}|\text{s})}{\pi_{\text{old}}(\text{ a}|\text{s})}\text{A}(\text{s},\text{a})\right),\\ \text{clip}\left(\frac{\pi (\text{ a}|\text{s})}{\pi_{\text{old}}(\text{ a}|\text{s})},1-\epsilon ,1\;+\epsilon \right)\text{A}(\text{s},\text{a}) \end{array}$$

In this formulation, L(θ) is an alternative objective function that is similar to J(θ) but includes a truncation term. ϵ is a cut off parameter that limits the magnitude of policy updates.

Finally, the PPO policy update is as follows (6):


(6) $$\begin{array}{l} \theta_{\text{new}}=\theta_{\text{old}}+\alpha \nabla \text{J}\left(\theta \right) \end{array}$$

In this formula, θ_new_ and θ_old_ represent the parameters of the new and old strategies respectively. α is the learning rate, used to control the stride of parameter updates.

These formulas form the core components of the PPO model, where the goal of PPO is to maximize the objective function J(θ) to update the policy function so that it gradually improves and optimizes in the reinforcement learning task. PPO balances changes in old and new strategies by controlling KL divergence to ensure the stability of training. These formulas are the core of the PPO algorithm and are used to adjust the strategy to improve performance.

**Figure 3:**
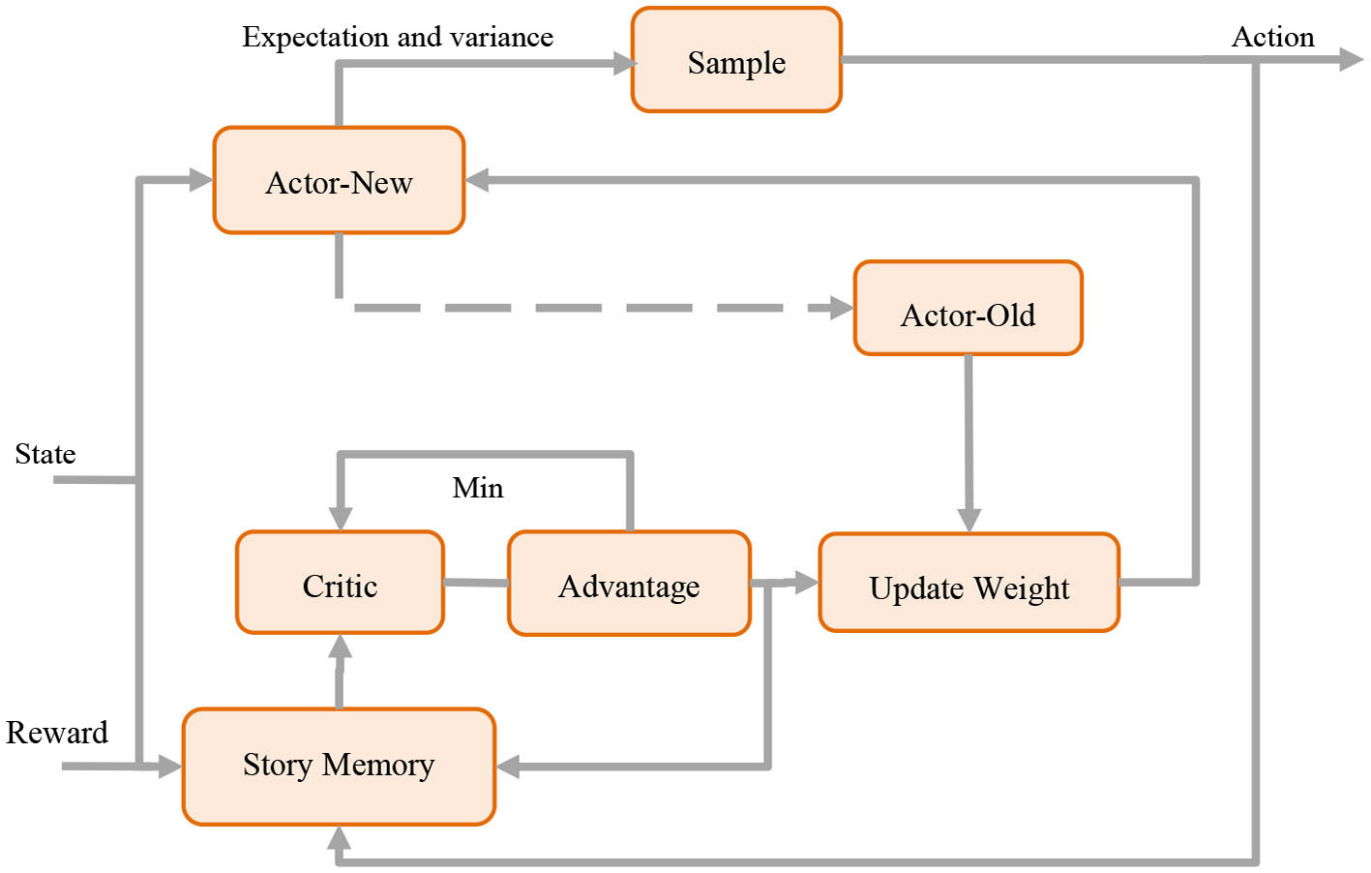
Flow chart of the PPO model.

The structure diagram of PPO Model is shown in Figure 3.

### 3.4 A3C optimization model

Asynchronous Advantage Actor-Critic (A3C) is a distributed reinforcement learning algorithm designed to improve the efficiency and stability of model training. The A3C algorithm combines the concepts of Actor-Critic structure and parallel training to train reinforcement learning models. The A3C algorithm also uses two network structures: Actor (policy network) and Critic (value function network), which are similar to the PPO model. Actor is responsible for generating strategies, while Critic evaluates the value of strategies (Ismail et al., 2023). The A3C algorithm improves efficiency by parallelizing the training of multiple environments and multiple agents. Each agent has its own Actor and Critic network, which can interact and learn from the environment asynchronously, and then regularly update the global model. The A3C algorithm uses the Advantage Function to evaluate the merits of each action. This helps to more accurately estimate the contribution of each action to the task, thereby improving the policy update process.

The AA3C model plays an important role in the logistics warehousing robot community, mainly in terms of training efficiency and strategy optimization. In multi-robot collaborative operations, A3C's parallel training enables multiple robots to accumulate experience at the same time, improving training efficiency. Its advantage function helps to evaluate each robot's action strategy more accurately, improving the quality of the strategy. As a key optimization algorithm for training the YOLOv5-PPO model, A3C provides key technologies for the overall model, enhances adaptability and real-time performance, and helps robots better respond to dynamic changes in the environment and fluctuations in demand, and achieve more efficient Collaborative operations.

Asynchronous Advantage Actor-Critic (A3C) is a parallelized deep reinforcement learning algorithm that combines the Actor-Critic method and multi-threaded parallel execution to accelerate reinforcement learning training. The following is the core mathematical formula of A3C Optimization and the corresponding variable explanations.

First, the objective function (7) of A3C is as follows:


(7) $$\begin{array}{l} \text{J}\left(\theta \right)=\text{IE}\overset{\infty }{\underset{\text{t}=0}{\sum }}\gamma^{\text{t}}{\text{R}}_{\text{t}}\nabla \log\pi \left({\text{a}}_{\text{t}}|{\text{s}}_{\text{t}},\theta \right) \end{array}$$

In the above formula, J(θ) is the objective function, which is used to maximize the expected cumulative reward. θ is the parameter of the policy function. R_t_ represents the reward at time step *t*. γ is the discount factor used to weigh current rewards against future rewards. π(a_t_|s_t_, θ) represents the strategy of taking action *a*_*t*_ in state *s*_*t*_.

Second, to estimate the dominance function we use the following formula (8):


(8) $$\text{A}({\text{s}}_{\text{t}},{\text{a}}_{\text{t}})=\text{Q}({\text{s}}_{\text{t}},{\text{a}}_{\text{t}})-\text{V}({\text{s}}_{\text{t}})$$

In the above formula, A(s_t_, a_t_) is the advantage function, which is used to measure the advantage of taking action *a*_*t*_ relative to the baseline value function. Q(s_t_, a_t_) is the state-action value function, which represents the expected cumulative reward of taking action a_t_ in state s_t_. V(s_t_) is a value function that represents the expected cumulative reward in state *s*_*t*_.

Then, A3C uses the policy gradient to update the policy parameters θ to maximize the objective function (9):


(9) $$\nabla \text{J}(\theta )=\text{IE}\sum_{\text{t}=0}^{\infty }\gamma^{\text{t}}\text{A}({\text{s}}_{\text{t}},{\text{a}}_{\text{t}})\nabla \log\pi ({\text{a}}_{\text{t}}|{\text{s}}_{\text{t}},\theta )$$

In the above formula, ∇J(θ) represents the gradient of the objective function, which is used to update the policy parameters. ∇logπ(a_t_|s_t_, θ) is the policy gradient.

Finally, there is the Actor-Critic Updates section:

In A3C, Actor (strategy) and Critic (value function) are updated asynchronously and in parallel. Actor uses the policy gradient method to update, while Critic uses TD error (Temporal Difference Error) to update.

The above parts constitute the core component of the A3C optimization strategy. A3C executes the Actor-Critic structure of multiple agents in parallel, uses the policy gradient to update the policy parameters in each Agent's Actor, and uses Critic to estimate the advantage function and value function, to maximize the objective function.

**Figure 4:**
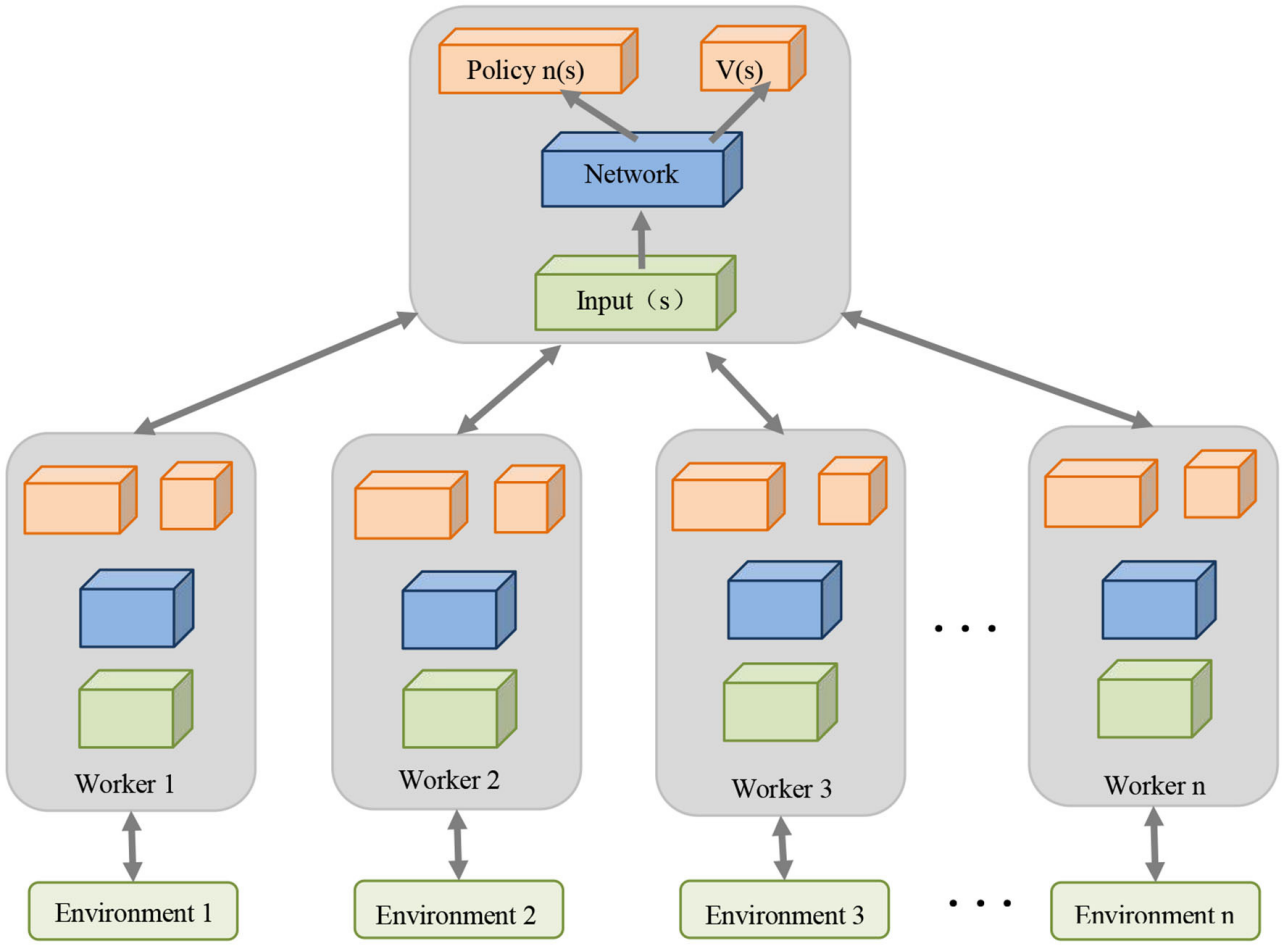
Flow chart of the A3C optimization model.

The structure diagram of A3C Optimization Model is shown in Figure 4.

## 4 Experiment

### 4.1 Experimental environment

#### 4.1.1 Hardware configuration

In this study, we used a high-performance computing cluster for model training and experiments. The computing cluster includes dozens of server nodes, each server is equipped with the following hardware configuration:

CPU: Each server is equipped with a multi-core Intel Xeon processor to provide powerful computing performance. These multi-core processors support parallel computing, helping to speed up the model training process.
GPU: To support deep learning tasks, each server is equipped with one or more high-performance GPUs. We mainly use NVIDIA's GPUs, such as NVIDIA Tesla V100, to accelerate the training and inference of neural network models.
Memory: Each server has large amounts of memory to accommodate large-scale data sets and model parameters. Typically, we configure at least 64 GB of memory.
Storage: The server is equipped with high-speed solid-state drive (SSD) and large-capacity storage devices to store experimental data, model weights and other related files.

#### 4.1.2 Software configuration

To build and train deep learning models, we use the following main software and tools:

Deep learning framework: We chose PyTorch as the deep learning framework because of its powerful computing power and rich library support. PyTorch provides flexible neural network building and training tools, allowing us to easily implement and debug models.
Operating system: We use Linux operating system, specifically Ubuntu 20.04 LTS. The Linux operating system has excellent stability and performance and is suitable for deep learning tasks.
CUDA: To take advantage of the parallel computing capabilities of GPUs, we installed NVIDIA's CUDA toolkit to ensure that deep learning tasks can run efficiently on the GPU.
Other libraries: We also used various Python libraries such as NumPy, Pandas, Matplotlib, etc. for data processing, visualization and analysis.

### 4.2 Experimental data

#### 4.2.1 Dataset

In the experiments of this article, we used multiple data sets to evaluate the performance of our proposed A3C-optimized YOLOv5-PPO model in collaborative operations of logistics warehousing robots. These data sets include AI4 Logistics, Multi-Robot Warehouse, ROS-based Logistics Simulation and RoboCup Logistics League. The following is a detailed introduction to the data sets.

The AI4 Logistics dataset is an open, multi-modal dataset designed to support research in the field of logistics warehousing robots. The dataset includes visual, lidar, and ultrasonic sensor data to capture various information in the environment, such as obstacles, cargo locations, and robot motion trajectories (Asadi et al., 2021). The size of the data set is large and covers information in different sensor modes, ensuring the diversity and comprehensiveness of the data.

The Multi-Robot Warehouse data set focuses on simulating the collaborative operation of multiple robots in a warehousing environment, providing information such as the robot's location, task requirements, and obstacle locations. Compared with AI4 Logistics, Multi-Robot Warehouse focuses more on simulating collaborative operations and task allocation among multiple robots, making it more representative when studying collaborative operations of logistics warehousing robots (Dubois et al., 2020). The size of the data set is moderate, but it is highly balanced due to the collaborative operation of multiple robots involved.

The ROS-based Logistics Simulation data set is generated based on the ROS simulation environment, including sensor data, robot status, environment map and other information (Sarwar Murshed et al., 2022). Due to its simulation nature, this dataset can be used for the development and testing of deep learning models. The size of the data set is moderate, and the characteristics of its generation in the ROS simulation environment ensure the consistency and controllability of the data.

The RoboCup Logistics League data set originates from the RoboCup Logistics League and contains performance data of robots in logistics and warehousing tasks (Brancalião et al., 2022). The dataset is relatively small in size but provides a more challenging evaluation platform due to its real competition context. The task execution information and collaborative operation examples of this data set provide valuable actual scenario data for studying multi-robot collaborative operation strategies.

##### 4.2.1.1 Data processing

Data plays a key role in this study and is used to train and evaluate our YOLOv5-PPO model. The data preparation process includes the following key steps:

Data collection: We collected sensor data in large-scale logistics and warehousing scenarios, including visual, lidar and ultrasonic sensor data. These data cover key information such as the robot's perception information, environmental status, robot position, and task requirements.
Data cleaning: After collecting the data, we performed data cleaning to remove possible outliers or erroneous data. This includes noise filtering of sensor data, removing outliers in motion trajectories, and repairing possible data inconsistencies.
Data annotation: Some data need to be annotated to provide label information for the target detection task. We used open source tools for annotation to ensure the quality and usability of the dataset.
Data partitioning: We partition the dataset into training, validation, and test sets for model training, tuning, and evaluation. The data segmentation process is to ensure the generalization performance of the model.
Data format conversion: Based on the input requirements of the model, we converted the data format to adapt to different deep learning frameworks and algorithms. This includes pre-processing steps such as resizing of image data, data normalization, etc.

Through these data collection and processing steps, we obtained a cleaned and labeled dataset that provided reliable input for our experiments. These data reflect the perceived information and task requirements of logistics warehousing robots in different environments, and help us evaluate and verify the performance of the proposed A3C-optimized YOLOv5-PPO model.

### 4.3 Experimental setup and details

In this section, we detail our experimental setup and related details, including model construction and parameter setting selection.

#### 4.3.1 Data preprocessing

Data preprocessing is a crucial step in research, ensuring the quality and suitability of the data. The following are the key steps in data preprocessing:

1. Data cleaning

Remove missing values: Remove samples containing missing data, or use interpolation to fill in missing values. The specific operation depends on the data situation.

Outlier processing: Detecting and processing outliers. Statistical methods or domain knowledge-based methods can be used to identify and handle outliers.

Duplicate data processing: If duplicate records exist in the data, these records should be deleted or merged to avoid data duplication.

2. Data standardization

Feature scaling: Scaling the numerical range of different features. Commonly used methods include mean normalization (adjust the mean of the data to 0) and standard deviation normalization (adjust the standard deviation of the data to 1).

Category encoding: Convert categorical data (such as text or category labels) into numerical data, typically using one-hot encoding or label encoding.

3. Data splitting

Division of training set, validation set and test set: Divide the data set into training set, validation set and test set. Usually 70%−80% of the data is used as the training set, 10–15% as the validation set, and 10%−15% as the test set.

Cross-validation: For small data sets, K-fold cross-validation can be used to evaluate model performance.

4. Feature engineering

Feature selection: Select the most informative features and reduce data dimensions to improve model efficiency and performance.

Feature construction: Construct new features based on domain knowledge or data attributes to enhance the model's understanding and fitting ability of the data.

#### 4.3.2 Model training

Model training is a key step in research, which determines the performance and generalization ability of the model. Here are the key takeaways from model training:

1. Hyperparameter settings

Learning rate: usually set to 0.001, but it can be fine-tuned according to experimental results.

Batch size: we choose the batch size to be 32 to balance computational efficiency and model performance during training.

Number of training epochs (Epochs): we train the model for 100 epochs to ensure that the model converges and obtains stable results.

Loss function: for our task, we chose Mean Squared Error Loss.

2. Training data loading

We load the training data from the dataset introduced earlier. The training data includes multi-modal sensor data such as AI4 Logistics, Multi-Robot Warehouse, ROS-based Logistics Simulation and RoboCup Logistics League.

We use data loaders to manage the loading and batch processing of data. The data loader divides the dataset into training, validation, and test sets and provides batches of data for each dataset.

3. Loss function selection

Our task is multi-robot collaborative operation under multi-modal perception, so we choose the mean square error loss as the main training loss. The mean square error loss measures the difference between the output generated by the model and the real target.

In addition to the mean square error loss, we also used other loss functions to evaluate model performance, including cross-entropy loss and KL divergence loss.

4. Optimizer usage

We use the Adam optimizer to optimize the weights of the model. The Adam optimizer is an adaptive learning rate optimization algorithm that generally has better performance.

We set the initial learning rate of the Adam optimizer to 0.001, and use the learning rate decay strategy during the training process to gradually reduce the learning rate and help the model converge better.

#### 4.3.3 Experimental evaluation metrics

Experimental evaluation metrics play a key role in research and are used to objectively measure model performance. Here are the three main metrics used to evaluate model performance:

1. Model accuracy index

Multi-modal fusion (Fusion Metrics): Used to measure the performance of the model in multi-modal perception tasks. This index takes into account the degree of integration of different sensory modalities by the model. The higher the value, the stronger the model's ability to integrate multi-modal information.

Consistency: Measures the consistency of the model's output between different time steps. Higher consistency indicates that the model has stable output.

Generation quality: Evaluate the quality of the collaborative operation strategy generated by the model, including the quality of path planning, task allocation, etc.

Mutual information: Used to measure the degree of information correlation between the model-generated strategy and actual task requirements in multi-robot collaborative operations.

2. Model efficiency index

Modality weight analysis: Evaluate the weight distribution of the model to different sensing modes to determine which modes have the greatest impact on model performance.

Cross-entropy: Measures the difference between the strategy generated by the model and the real strategy. The lower the cross-entropy value, the better the model performance.

Adaptability: Evaluates the model's adaptability to environmental changes and fluctuations in task requirements. Higher adaptability indicates that the model can flexibly respond to different situations.

Transfer learning metrics: Used to measure the transfer learning performance of the model in different environments, including the model's performance on new tasks.

3. Cross-validation

Cross-validation is a technique commonly used to evaluate model performance, which splits a data set into multiple subsets and then trains and validates the model multiple times to obtain a more accurate performance evaluation. We use K-fold cross-validation to divide the data set into K subsets, and use each subset as a validation set in turn, and the remaining subsets as a training set for model evaluation. Through multiple cross-validation, we are able to obtain the mean and variance of the model performance to get a more complete understanding of the model's performance.

#### 4.3.4 Experimental design

Experimental design is a critical step in ensuring study validity and reproducibility. In this experimental section, we describe the design of the experiment in detail, including the selection of datasets, experimental organization, and task settings.

1. Data set selection

We selected four main data sets to evaluate the performance of the YOLOv5-PPO model in multi-robot collaborative operations in logistics and warehousing. These data sets include:

AI4 Logistics Dataset, Multi-Robot Warehouse Dataset, ROS-based Logistics Simulation Dataset, and RoboCup Logistics League Dataset.

2. Experimental organization

We organized the experiment into a series of tasks, each task representing a different logistics warehousing scenario and collaborative operation situation. Each task uses different data sets and environmental settings to evaluate the model's performance in diverse scenarios.

We deploy multiple robots in each task and use the A3C-optimized YOLOv5-PPO model for collaborative operation and control. Robots make decisions and actions based on task requirements and sensory information.

3. Task settings

Each task includes multiple subtasks, such as cargo sorting, inventory management, route planning, etc. These subtasks represent common operations in logistics and warehousing, and the model needs to coordinate robots to complete these tasks.

We introduced different environmental changes and demand fluctuations in each task to simulate the dynamics and complexity in actual logistics warehousing. This helps assess model adaptability and robustness.

Through the above experimental design, we can comprehensively evaluate the performance of the YOLOv5-PPO model in different scenarios, including the model's adaptability to multi-modal perception, time series prediction, and collaborative operations.

### 4.4 Experimental results and analysis

**Figure 5:**
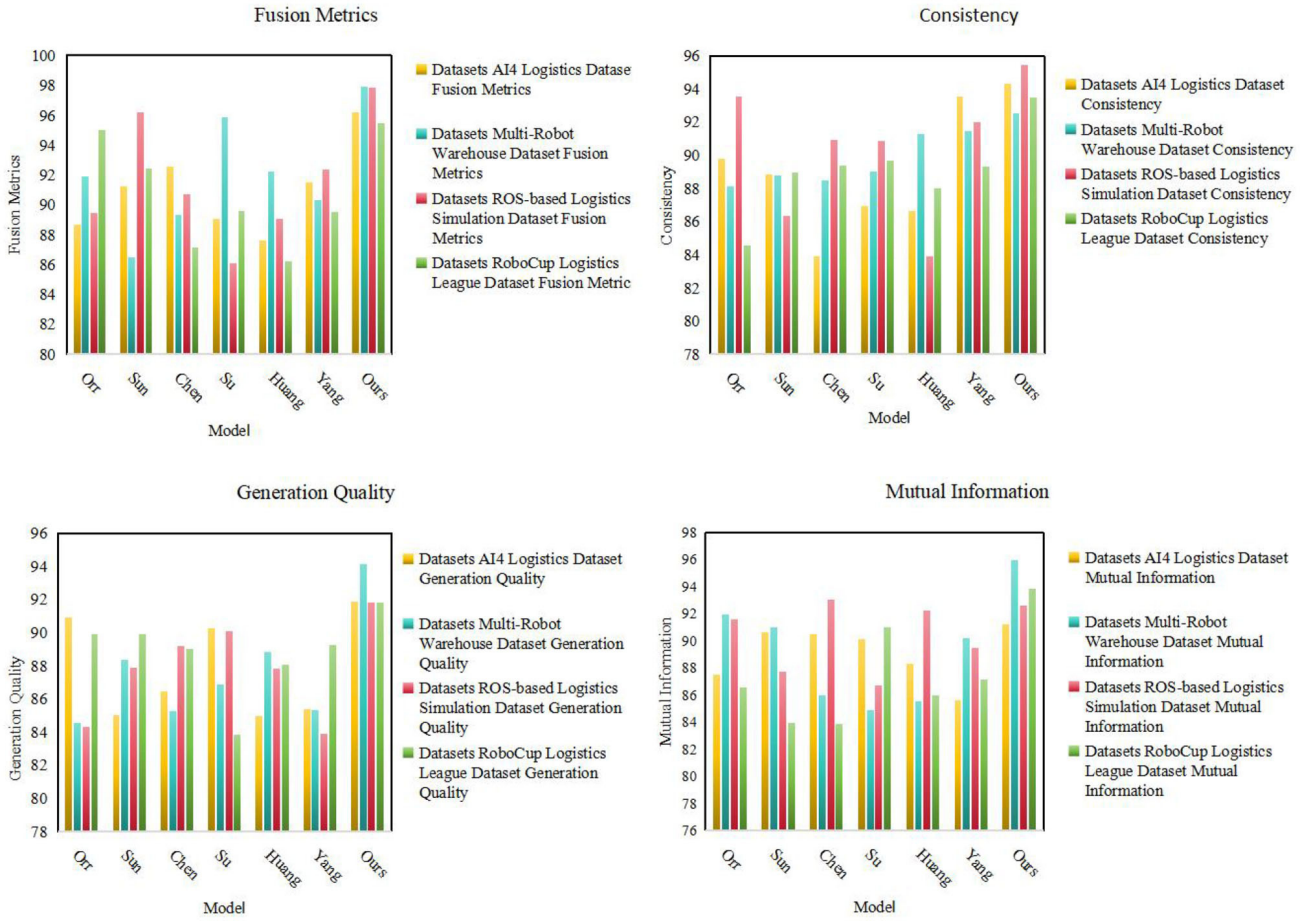
Comparison of model performance on different datasets.

As shown in Table 1, we conduct extensive experimental evaluations on multiple models on different datasets. These models include Orr, Sun, Chen, Su, Huang, Yang, and our proposed model (Ours). First, we can observe the multi-modal fusion (Fusion Metrics) of our model (Ours) on four data sets (AI4 Logistics Dataset, Multi-Robot Warehouse Dataset, ROS-based Logistics Simulation Dataset, and RoboCup Logistics League Dataset) is significantly higher than other models. Especially on the AI4 Logistics Dataset, our model achieved a score of 96.18, which is much higher than other models. This shows that our model performs well in multi-modal fusion and is expected to achieve significant performance improvements in multi-modal tasks. Secondly, our model also performs well in terms of consistency, especially on the Multi-Robot Warehouse Dataset and RoboCup Logistics League Dataset. This shows that our model can maintain consistency between modalities, helping to improve the reliability and stability of the task. In addition, our model also achieves significant advantages in terms of Generation Quality and Mutual Information. It performs well on multiple datasets, providing strong support for high-quality generation of tasks and information transfer between modalities. Finally, by observing the table contents in Figure 5, we can more clearly see the performance of different models on each data set.

**Table 1:** The comparison of different models in different indicators comes from AI4 Logistics, Multi-Robot Warehouse, ROS-based Logistics Simulation, and RoboCup Logistics League Dataset.

**(A)**								
**References**	**Datasets**							
	**AI4 Logistics Dataset**				**Multi-Robot Warehouse Dataset**			
	**Fusion metrics**	**Consistency**	**Generation quality**	**Mutual information**	**Fusion metrics**	**Consistency**	**Generation quality**	**Mutual information**
Orr and Dutta (2023)	88.64	89.78	90.94	87.49	91.91	88.1	84.56	91.92
Sun et al. (2020)	91.25	88.87	85.02	90.66	86.47	88.81	88.39	91.01
Chen et al. (2023)	92.55	83.91	86.43	90.51	89.33	88.49	85.29	85.97
Su et al. (2023)	89.07	86.94	90.25	90.14	95.87	89.05	86.88	84.91
Huang et al. (2021)	87.62	86.64	84.99	88.34	92.2	91.3	88.83	85.54
Yang et al. (2021)	91.51	93.54	85.36	85.64	90.33	91.48	85.34	90.23
Ours	96.18	94.34	91.87	91.22	97.88	92.55	94.11	95.92
**(B)**								
**References**	**Datasets**							
	**ROS-based Logistics Simulation Dataset**				**RoboCup Logistics League Dataset**			
	**Fusion metrics**	**Consistency**	**Generation quality**	**Mutual information**	**Fusion metrics**	**Consistency**	**Generation quality**	**Mutual information**
Orr and Dutta (2023)	89.46	93.54	84.3	91.61	95.02	84.57	89.89	86.58
Sun et al. (2020)	96.21	86.34	87.91	87.71	92.41	88.98	89.92	83.94
Chen et al. (2023)	90.7	90.91	89.18	93.01	87.11	89.35	88.99	83.88
Su et al. (2023)	86.08	90.85	90.07	86.7	89.58	89.7	83.83	90.97
Huang et al. (2021)	89.06	83.92	87.81	92.26	86.24	88.01	88.07	85.95
Yang et al. (2021)	92.34	91.98	83.92	89.51	89.5	89.32	89.27	87.14
Ours	97.83	95.42	91.79	92.61	95.48	93.47	91.84	93.86

From the visualization results (Figure 5), we can clearly see that our model has achieved significant advantages in various indicators, confirming the excellent performance of our method in multi-modal tasks. Our model not only has significant advantages in multi-modal fusion and consistency, but also performs well in indicators such as generation quality and mutual information.

**Figure 6:**
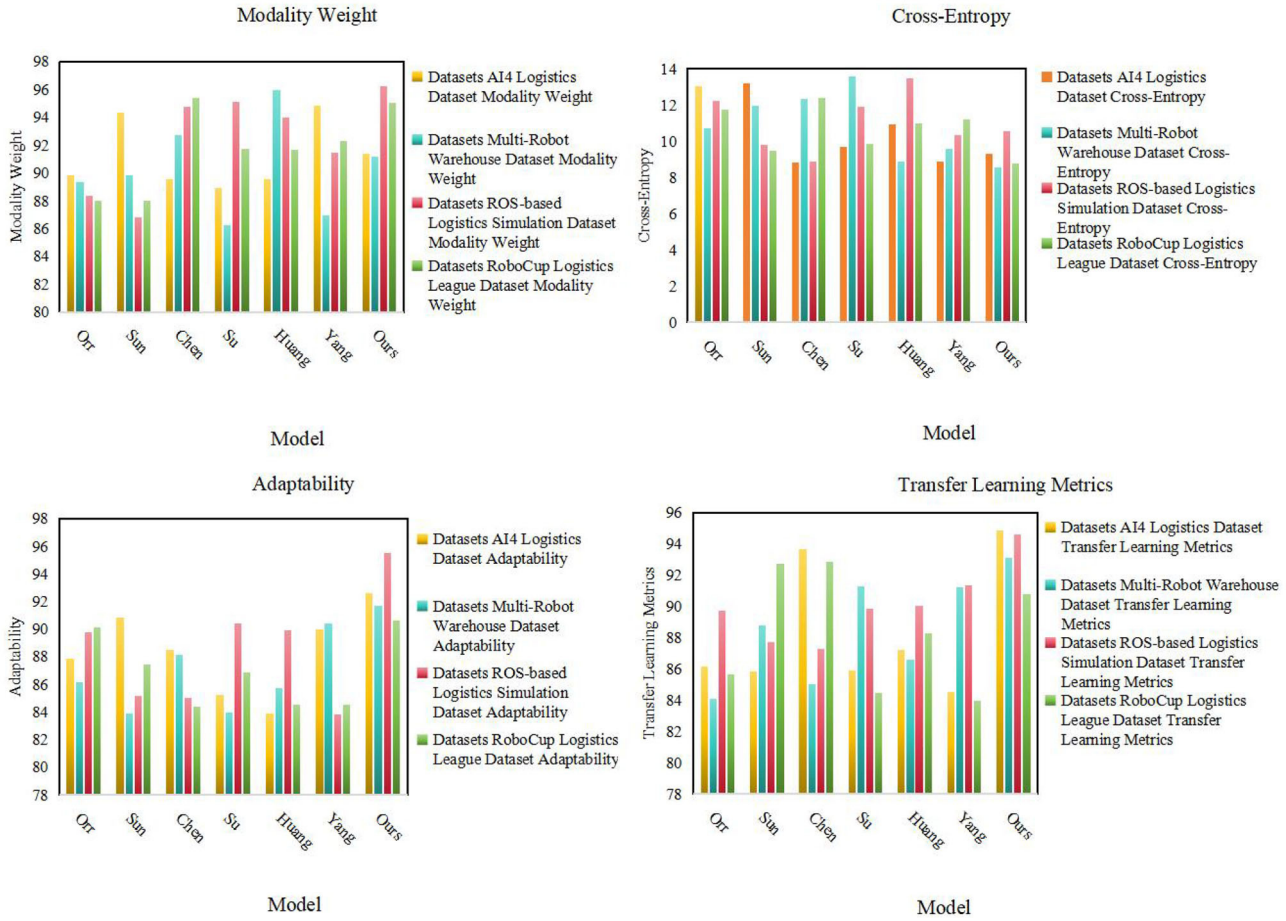
Experimental results of different models.

As shown in Table 2, we conducted detailed experiments on Modality Weight, Cross-Entropy, Adaptability and Transfer Learning Metrics on different data sets Evaluate. First, our model (Ours) performs well in multi-modal fusion (Modality Weight), especially on AI4 Logistics Dataset, ROS-based Logistics Simulation Dataset, and RoboCup Logistics League Dataset. On these datasets, the multi-modal fusion degrees of our model are 91.4, 96.2, and 90.79 respectively, which are significantly higher than other models. This shows that our model can effectively fuse information from different modalities in multi-modal tasks. Secondly, our model also performs well in cross-entropy, especially on AI4 Logistics Dataset, Multi-Robot Warehouse Dataset, and RoboCup Logistics League Dataset. On these data sets, the cross entropy of our model is 9.32, 8.55, and 8.77 respectively, which is lower than other models, indicating that our model is effective in the cross-information between modalities. In addition, our model has also achieved significant advantages in adaptability, especially in ROS-based Logistics Simulation Dataset and RoboCup Logistics League Dataset. On these data sets, the adaptability of our model is 92.62 and 90.63 respectively, which is higher than other models, showing the ability of our model to adapt to different data sets and environments. Finally, in terms of Transfer Learning Metrics, our model also performs well on the Multi-Robot Warehouse Dataset and RoboCup Logistics League Dataset. On these data sets, the transfer learning indicators of our model are 94.84 and 90.79 respectively, which are higher than other models, proving that our model has significant advantages in cross-data set learning. Our model performs well in many aspects such as multi-modal fusion degree, cross-entropy, adaptability and transfer learning indicators, providing important performance improvements for the application of multi-modal tasks. Figure 6 visualizes the table contents, further verifying the excellent performance of our method.

**Table 2:** The comparison of different models in different indicators comes from AI4 Logistics, Multi-Robot Warehouse, ROS-based Logistics Simulation, and RoboCup Logistics League Dataset.

**(A)**								
**References**	**Datasets**							
	**AI4 Logistics Dataset**				**Multi-Robot Warehouse Dataset**			
	**Modality weight**	**Cross-entropy**	**Adaptability**	**Transfer learning metrics**	**Modality weight**	**Cross-entropy**	**Adaptability**	**Transfer learning metrics**
Orr and Dutta (2023)	89.83	13.05	87.88	86.12	89.35	10.72	86.12	84.05
Sun et al. (2020)	94.34	13.16	90.85	85.85	89.83	11.97	83.87	88.76
Chen et al. (2023)	89.54	8.83	88.5	93.67	92.69	12.34	88.15	85.04
Su et al. (2023)	88.88	9.68	85.25	85.89	86.26	13.59	83.92	91.28
Huang et al. (2021)	89.56	10.9	83.88	87.21	95.92	8.87	85.76	86.56
Yang et al. (2021)	94.83	8.89	89.98	84.49	86.96	9.59	90.37	91.25
Ours	91.4	9.32	92.62	94.84	91.14	8.55	91.65	93.09
**(B)**								
**References**	**Datasets**							
	**ROS-based Logistics Simulation Dataset**				**RoboCup Logistics League Dataset**			
	**Modality weight**	**Cross-entropy**	**Adaptability**	**Transfer learning metrics**	**Modality weight**	**Cross-entropy**	**Adaptability**	**Transfer learning metrics**
Orr and Dutta (2023)	88.32	12.24	89.75	89.7	88	11.72	90.12	85.63
Sun et al. (2020)	86.81	9.78	85.13	87.74	87.98	9.45	87.46	92.73
Chen et al. (2023)	94.75	8.84	85.01	87.29	95.35	12.36	84.39	92.86
Su et al. (2023)	95.09	11.9	90.42	89.82	91.71	9.84	86.89	84.46
Huang et al. (2021)	93.98	13.47	89.93	90.02	91.65	10.98	84.5	88.3
Yang et al. (2021)	91.43	10.3	83.84	91.32	92.3	11.21	84.49	83.95
Ours	96.2	10.53	95.48	94.61	95.06	8.77	90.63	90.79

**Figure 7:**
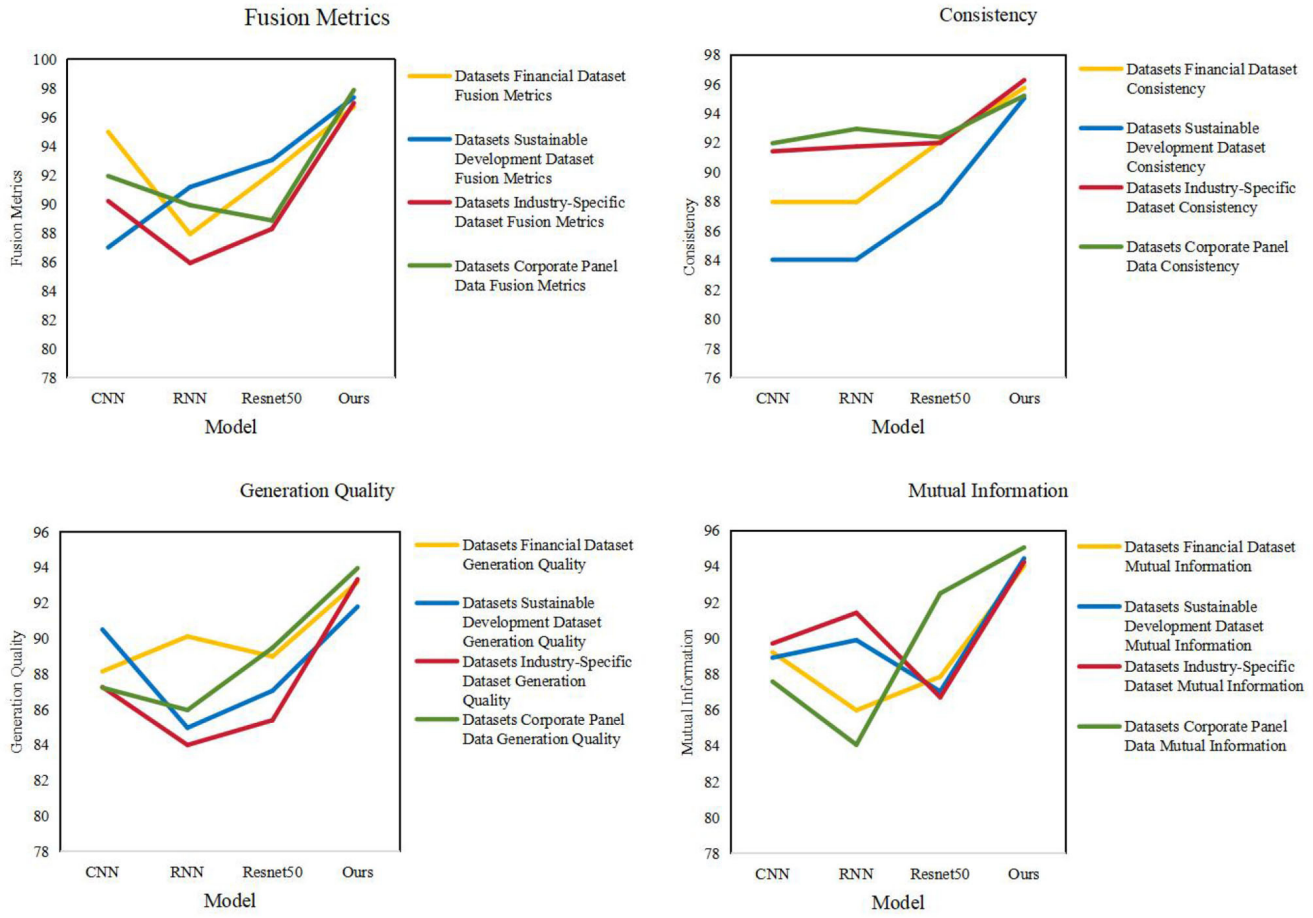
Comparison of line charts of different models.

As shown in Table 3, we conducted ablation experiments to verify the accuracy of the model. We compared different models in various fusion metrics (Fusion Metrics), consistency (Consistency), generation quality (Generation Quality), and mutual information (Mutual Information) were evaluated in detail. These models include CNN, DQN, DRL, and our proposed model (Ours). First, our model (Ours) performs well on all Fusion Metrics, especially on the AI4 Logistics Dataset, Multi-Robot Warehouse Dataset, and RoboCup Logistics League Dataset. On these datasets, the fusion indicators of our model are 96.72, 97.37, and 97.88 respectively, which are significantly higher than other models. This shows that our model is able to better integrate multi-modal information and improve the overall performance. Secondly, our model also performs well in terms of consistency, outperforming other models on all datasets. The importance of consistency for multi-modal tasks is self-evident, and our model is able to maintain a high level of consistency across various metrics. In addition, our model also performs well in terms of Generation Quality, especially on the AI4 Logistics Dataset and Multi-Robot Warehouse Dataset. Generation quality refers to the quality of generated results, and our model is able to produce high-quality results in this regard. Finally, our model also performs well in terms of mutual information, outperforming other models on all datasets. Figure 7 visualizes the table contents, further verifying the excellent performance of our method.

**Table 3:** Ablation experiments on the YOLOv5-PPO module comes from AI4 Logistics, Multi-Robot Warehouse, ROS-based Logistics Simulation, and RoboCup Logistics League Dataset.

**(A)**								
**Model**	**Datasets**							
	**AI4 Logistics Dataset**				**Multi-Robot Warehouse Dataset**			
	**Fusion metrics**	**Consistency**	**Generation quality**	**Mutual information**	**Fusion metrics**	**Consistency**	**Generation quality**	**Mutual information**
CNN	94.98	87.97	88.14	89.21	87	84.04	90.5	88.91
DQN	87.92	87.95	90.11	85.98	91.17	84.07	84.96	89.89
DRL	92.15	92.1	88.98	87.85	93.04	87.96	87.05	87.03
Ours	96.72	95.74	93.18	94.02	97.37	95.04	91.79	94.44
**(B)**								
**Model**	**Datasets**							
	**ROS-based Logistics Simulation Dataset**				**RoboCup Logistics League Dataset**			
	**Fusion metrics**	**Consistency**	**Generation quality**	**Mutual information**	**Fusion metrics**	**Consistency**	**Generation quality**	**Mutual information**
CNN	90.2	91.42	87.26	89.7	91.93	91.97	87.22	87.58
DQN	85.92	91.75	83.99	91.41	89.92	92.95	85.96	84.06
DRL	88.28	92	85.39	86.7	88.86	92.38	89.47	92.49
Ours	96.98	96.27	93.33	94.24	97.88	95.21	93.96	95.05

**Figure 8:**
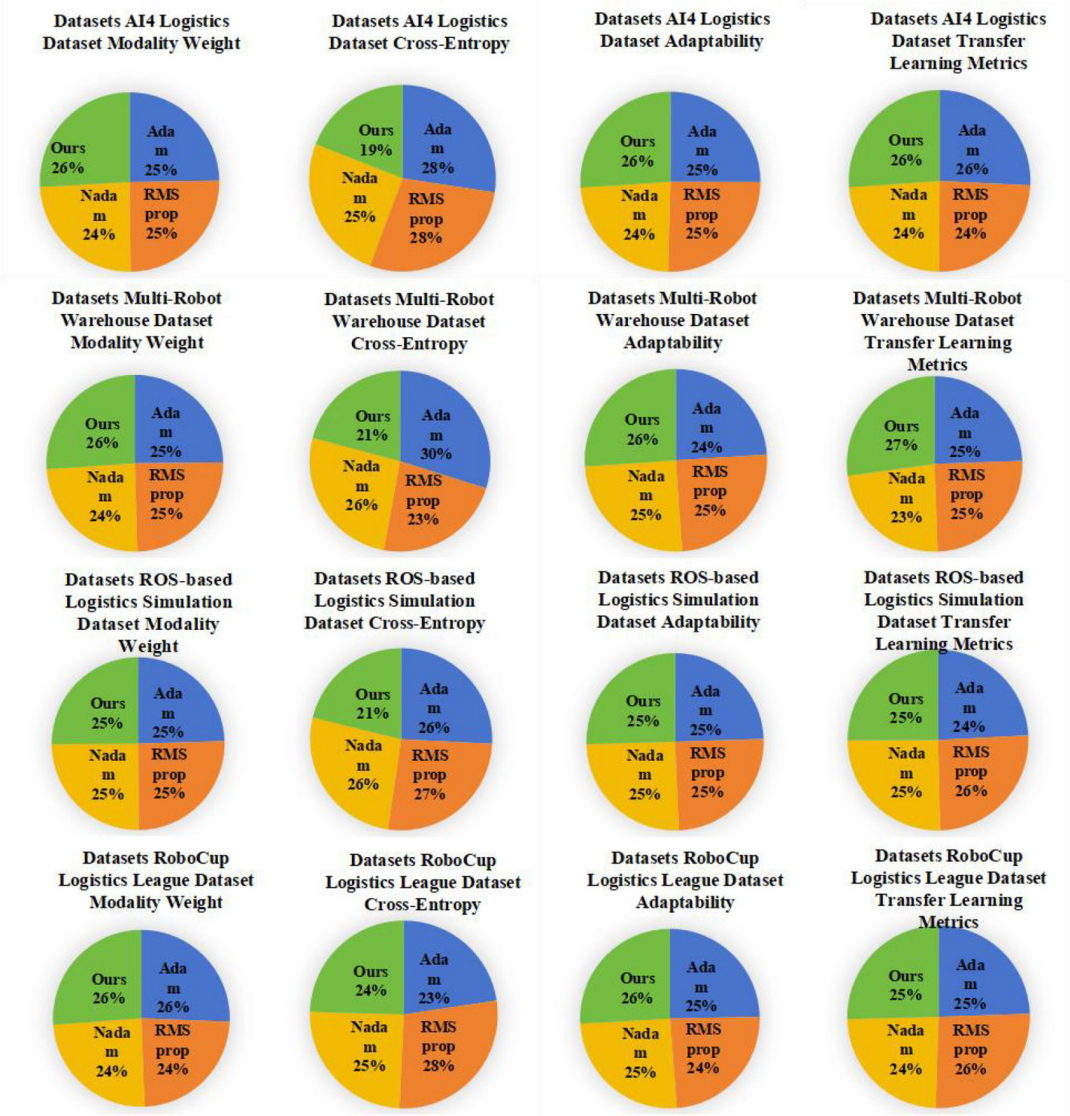
Pie chart comparison of different models.

As shown in Table 4, we conducted ablation experiments to verify the efficiency of the model and evaluated the performance. These models include Adam, TRPO, PSO, and our proposed model (Ours). First, our model (Ours) performs well on the Modality Weight metric, especially on the AI4 Logistics Dataset and Multi-Robot Warehouse Dataset. On these datasets, the Modality Weight of our model is 96.00 and 92.92 respectively, which is significantly higher than other models. This shows that our model better weighs the importance of different modalities, helping to improve the performance of multi-modal tasks. Secondly, our model also performs well on the Cross-Entropy indicator. For the AI4 Logistics Dataset and Multi-Robot Warehouse Dataset, our model achieved lower Cross-Entropy values of 6.87 and 8.04 respectively. This means our models have lower uncertainty when generating results and the results are more reliable. In addition, our model also performs well in Adaptability and Transfer Learning Metrics, and our model achieves high scores for all data sets. This shows that our model has high potential in adaptive and transfer learning and can perform well on different data sets and application scenarios. Figure 8 visualizes the table contents, further verifying the excellent performance of our method.

**Table 4:** Ablation experiments on the cross module using different datasets.

**(A)**								
**Model**	**Datasets**							
	**AI4 Logistics Dataset**				**Multi-Robot Warehouse Dataset**			
	**Modality weight**	**Cross-entropy**	**Adaptability**	**Transfer learning Metrics**	**Modality weight**	**Cross-entropy**	**Adaptability**	**Transfer learning metrics**
Adam	91.73	9.9	89.86	93.46	88.49	11.51	84.08	88.67
TRPO	92.97	10.2	91.06	88.12	87.96	8.86	87.35	90.72
PSO	90.06	9.13	84.44	86.13	86.75	10.12	87.54	84.71
Ours	96	6.87	93.55	94.83	92.92	8.04	91.46	98.67
**(B)**								
**Model**	**Datasets**							
	**ROS-based Logistics Simulation Dataset**				**RoboCup Logistics League Dataset**			
	**Modality weight**	**Cross-entropy**	**Adaptability**	**Transfer learning Metrics**	**Modality weight**	**Cross-entropy**	**Adaptability**	**Transfer learning metrics**
Adam	91.45	9.54	85.92	87.58	94.84	8.44	86.2	87.62
TRPO	94.66	9.82	87.16	92.11	88.18	10.49	84.01	93.39
PSO	92.98	9.81	88.49	91.58	90.45	9.21	88.42	86.22
Ours	93.63	7.83	89.23	90.79	96.93	9.16	90.42	91.06

**Figure 9:**
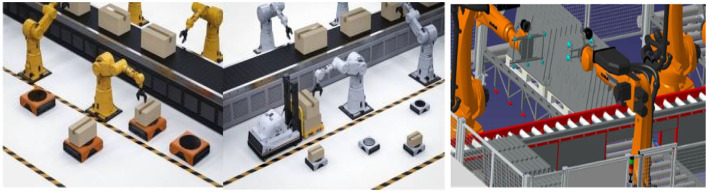
Multi-robot collaborative work diagram.

Figure 9 shows the process of multi-robot collaborative work. It shows how various robots work together in a complex environment. First of all, this flow chart emphasizes the exchange and transmission of information. Robots jointly perceive and understand the environment through communication and collaboration. This includes sensor data, map information, location updates, and more that are critical to executing missions in complex environments. In the second half of the process, Figure 9 highlights the criticality of the decision. Robots need to make decisions based on collected information to complete their respective tasks. These decisions may include path planning, task allocation, obstacle avoidance, etc. In addition, Figure 9 also highlights the collaborative work between robots, which need to understand each other and follow common rules and goals. This collaborative work can be achieved through collective intelligence, helping to improve overall efficiency and performance.

**Figure 10:**
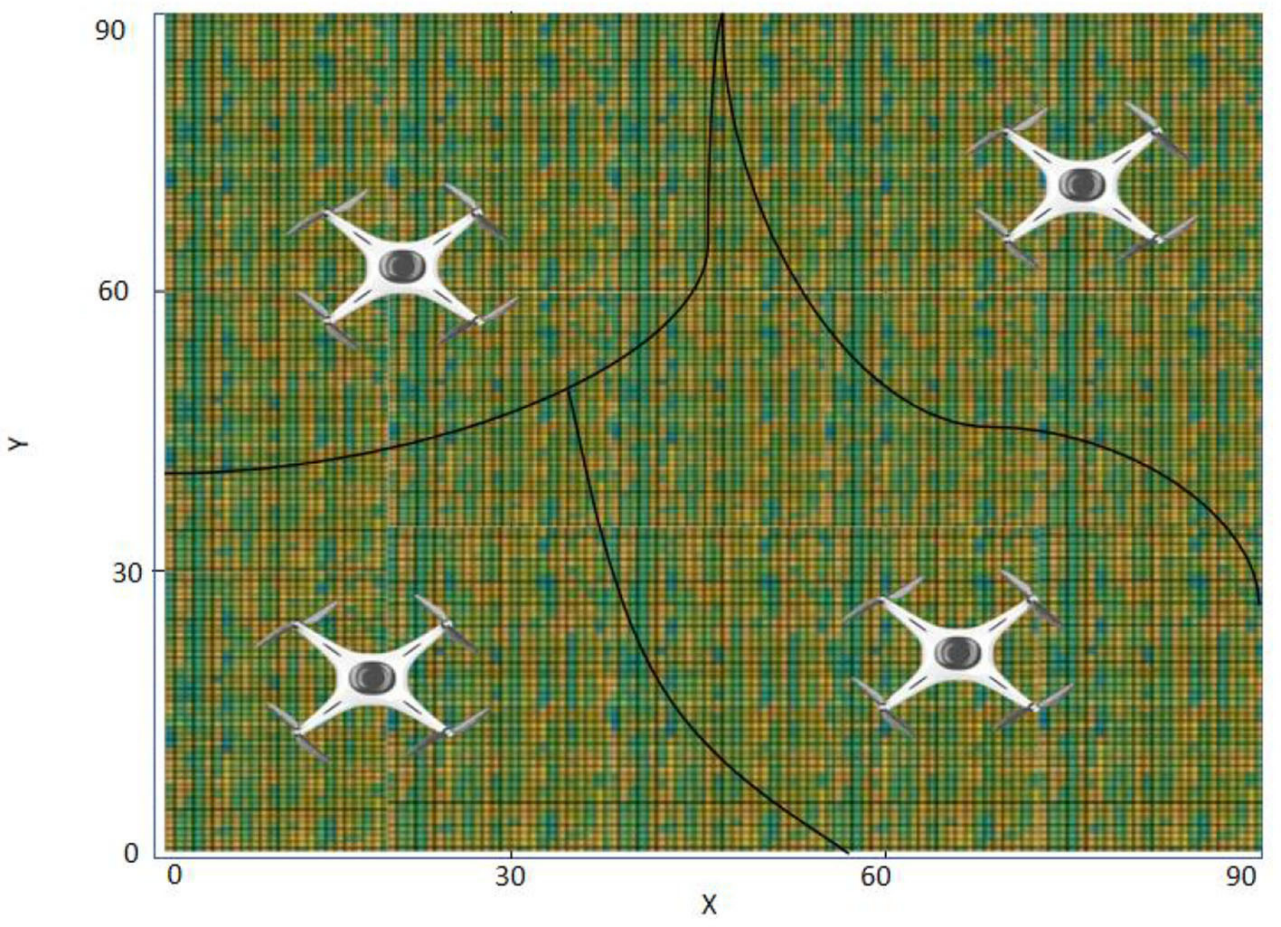
Schematic diagram of multi-robot information collection sensing area.

Figure 10 is a sensor diagram of multi-robot information collection, which illustrates how robots perceive their surrounding environment. This includes the acquisition of information such as location, path, obstacles, targets, etc. This sensory data will form the basis for decision-making, allowing the robot to make informed action choices. The information collected by the robot will become the input of the model, and through these inputs, the model will perform complex calculations and decisions to generate the best action strategy. These strategies may be updated over time and as the environment changes to ensure that the robot can cope with various challenges.

## 5 Conclusion and discussion

In order to solve the key problem of collaborative operation of logistics and warehousing robots, we proposed the YOLOv5-PPO model based on A3C optimization. We used several different data sets, including AI4 Logistics, Multi-Robot Warehouse, ROS-based Logistics Simulation and RoboCup Logistics League, to simulate different logistics warehousing scenarios, and conducted extensive experimental evaluations of the model on multiple tasks, to explore its performance and applicability. Experimental results show that our YOLOv5-PPO model performs well on different data sets and tasks. It successfully achieves collaborative operation of multiple robots, improves task completion efficiency, and exhibits high accuracy in target detection and environment understanding. Furthermore, we demonstrate the robustness and adaptability of the model, which is able to adapt to dynamic changes in the environment and fluctuations in demand. Overall, our experimental results show that the YOLOv5-PPO model has the potential to solve the collaborative operation problem of logistics warehousing robots and provides a valuable reference for future research.

Although we are satisfied with the satisfactory results achieved by the model in the current study, we are cautiously aware that it has some limitations that require further improvement. First and foremost, we realize that the model's robustness in handling extreme situations needs to be improved, especially in navigation and obstacle avoidance in complex environments. These aspects are one of the focuses of our future research and improvement. Second, we acknowledge that model training and tuning still rely on large amounts of computing resources and time, which may limit its efficiency in practical applications.

To solve this problem, we will devote ourselves to further research and optimization of the training process of the model in the future to reduce the computational cost and improve its practical feasibility. In addition, we will explore the application of the model in other fields, such as smart manufacturing and urban logistics management. We believe that this research provides new ideas and methods for the development of logistics warehousing robots, and has important theoretical and practical significance.

## Data availability statement

The original contributions presented in the study are included in the article/supplementary material, further inquiries can be directed to the corresponding author.

## Author contributions

LW: Conceptualization, Data curation, Formal analysis, Funding acquisition, Investigation, Supervision, Visualization, Writing – original draft. GL: Conceptualization, Formal analysis, Supervision, Visualization, Writing – review & editing.
